# Treatment with nintedanib is as effective and safe in patients with other connective tissue diseases (CTDs)-interstitial lung disease (ILD) as in patients with systemic sclerosis-ILD: A multicenter retrospective study

**DOI:** 10.1007/s10067-025-07323-0

**Published:** 2025-02-07

**Authors:** Salim Mısırcı, Ali Ekin, Burcu Yağız, Belkıs Nihan Coşkun, Fatma Başıbüyük, Ahmet Merih Birlik, İsmail Sarı, Aylin Dolu Karaca, Süleyman Serdar Koca, Gözde Yıldırım Çetin, Burak Okyar, Nurhan Atilla, Ediz Dalkılıç, Yavuz Pehlivan

**Affiliations:** 1 Department of Internal Medicine, Division of Rheumatology, Faculty of Medicine, Bursa Uludag University, Bursa, Turkey; 2 Department of Internal Medicine, Division of Rheumatology, Dokuz Eylul University Faculty of Medicine, İzmir, Turkey; 3 Department of Internal Medicine, Division of Rheumatology, Faculty of Medicine, Fırat University, Elazığ, Turkey; 4 Department of Internal Medicine, Division of Rheumatology, Faculty of Medicine, Kahramanmaras Sutcu Imam University, Kahramanmaras, Turkey; 5 Adana City Training and Research Hospital, Rheumatology, Adana, Turkey; 6 Department of Chest Diseases, Faculty of Medicine, Kahramanmaras Sutcu Imam University, Kahramanmaras, Turkey

**Keywords:** Connective tissue disease, Interstitial lung disease, Nintedanib, Real-world data, Systemic sclerosis

## Abstract

**Objective:**

The aim of this study is to assess the efficacy and safety of nintedanib (NTD) therapy in a real world population of patients with connective tissue diseases related interstitial lung disease (CTDs-ILD).

**Methods:**

Our multicenter retrospective study included patients with a CTD-ILD diagnosis who started NTD treatment due to the development of PPF during follow-up. The results of the percentage predicted forced vital capacity (%pFVC) and percentage predicted diffusion capacity (%pDLCO) of patients before NTD treatment and 6, 12 and 18 months after the start of NTD treatment were evaluated. In addition, the patients were divided into two groups, SSc-ILD and other CTDs-ILD, and compared in terms of the efficacy and safety of the NTD.

**Results:**

In all patients (*n* = 66), %pFVC and %pDLCO values stabilised after 6, 12 and 18 months compared to baseline values. All patients received at least one immunosuppressive therapy in combination with NTD treatment. The most common side effect after NTD treatment was diarrhoea (*n* = 20, 30.3%). When we divided the patients into two groups, SSc-ILD (*n* = 35) and other CTDs-ILDs (*n *= 31), no significant difference was found between the groups in the change in %pFVC (*p* values = 0.498, 0.595 and 0.376, respectively) and in the change in %pDLCO (*p* values = 0.817, 0.185 and 0.399, respectively) at 6, 12 and 18 months follow-up. Again, there was no statistically significant difference between the two groups in terms of adverse events and safety data after NTD treatment (*p* > 0.05).

**Conclusion:**

In summary, the use of NTD in combination with immunosuppressive therapies was effective and safe in SSc-ILD patients as well as in other CTDs-ILD patients.

**Table:** 

**Key Points** • *This multicenter study provides real-world data on the use of nintedanib in patients with connective tissue disease-interstitial lung disease.* • *Nintedanib treatment is as effective and safe in patients with other connective tissue disease as in patients with systemic sclerosis.*

## Introduction

Interstitial lung disease (ILD) is a clinical condition that can occur in association with connective tissue diseases (CTDs). It is characterised by inflammation and fibrosis of the lung parenchyma and increases mortality and morbidity during the course of the disease [1, 2].

ILD can occur with varying frequency in the course of CTDs such as systemic sclerosis (SSc), rheumatoid arthritis (RA), Sjögren's syndrome (SJS), mixed connective tissue disease (MCTD), undifferentiated connective tissue disease (UCTD) and idiopathic inflammatory myositis (IIM) [2].

During the course of SSc, ILD is detected in 63–65% of patients evaluated with high-resolution computed tomography (HRCT), whereas the rate of ILD detected with various diagnostic tools during the course of RA is 10% [3, 4]. In the course of SJS, it can occur in 10–27%, especially in patients with primary SJS [4].

In terms of patterns of involvement, the most common pattern of ILD in the course of RA is usual interstitial pneumonia (UIP), whereas the most common pattern of ILD in the course of other connective tissue diseases is non-specific interstitial pneumonia (NSIP) [4]. In the course of RA, progressive fibrosing ILD can be detected in about 55% of patients with UIP pattern, whereas it can be detected in about 30.8% of patients with NSIP pattern (progressive fibrotic NSIP) [5]. The pathogenesis of ILD in the course of CTD is complex, and immune-mediated lung inflammation is known to be important in the development and progression of ILD. However, the mechanisms leading to fibrosis are not clear [6]. The frequency with which patients with CTD-ILD develop a progressive pulmonary fibrosis (PPF) phenotype varies depending on the underlying disease [7].

Glucocorticoids (GCs), azathiopurine (AZT), mycophenolate mofetil (MMF), calcineurin inhibitors, cyclophosphamide (CYC), rituximab (RTX), tocilizumab (TCZ) and abatacept (ABT) can be used as immunosuppressive treatment for CTD-ILD [8, 9]. However, nintedanib (NTD), an antifibrotic drug, has come to the forefront in ILD with fibrosis that progresses despite current immunosuppressive therapies. NTD is a tyrosine kinase inhibitor that binds to the intracellular ATP-binding site of fibroblast growth factor receptors, platelet-derived growth factor receptors and vascular endothelial growth factor receptors, causing autophosphorylation of the receptors and blocking the signalling pathway [10].

In phase III clinical trials with NTD for the treatment of idiopathic pulmonary fibrosis (INPULSIS-1 and INPULSIS-2), NTD was shown to reduce the decline in percentage predicted forced vital capacity (%pFVC) and has become a new treatment option [11].

The INBUILD study in patients with progressive fibrosing-ILD not suffering from idiopathic pulmonary fibrosis, including CTD-ILD patients, and the SENSCIS study in patients with SSc-ILD also emphasised that NTD is effective and safe [12, 13].

In our study, we aimed to evaluate the efficacy and safety of NTD treatment in a real world population with CTDs-ILD.

## Methods

Our multicenter study (four rheumatology clinics) included patients with a CTD-ILD diagnosis who started NTD treatment due to the development of PPF during follow-up. After approval by the local ethics committee (April 3, 2024, 2024–5/16), patients were restrospectively scanned from the hospital registration system. In CTD-ILD patients with radiologic evidence of pulmonary fibrosis as PPF criterion; the presence of at least two of the following criteria was required.

1. worsening of respiratory symptoms in the last year,
2. physiologic evidence of disease progression (≥ 5% absolute decrease in %pFVC), 10% absolute decrease in percentage predicted diffusing capacity (%pDLCO)),
3. radiologic evidence of disease progression [14].

Patients without PFTs in the last 6 months before starting NTD treatment and at 6, 12 and 18 months during follow-up were excluded. Patients with other chest diseases or restrictive lung pathologies were also excluded. Demographic characteristics, CTD type (SSc, RA, SJS, MCTD, IIM, UCTD), comorbidities, immunosuppressive therapies used before and after NTD treatment (GCs, AZT, MMF, CYC, RTX, TCZ, ABT), disease duration before PPF and PPF progression criteria were determined and recorded. The patterns of involvement (UIP, probable UIP, fibrotic NSIP) of patients radiologically examined with HRCT were recorded.

The PFTs results (%pFVC, %pDLCO) of all patients before, 6, 12 and 18 months after the start of the NTD and the changes during follow-up were recorded and compared. A separate analysis was also performed for the follow-up of SSc patients.

Adverse events that occurred after NTD treatment and their severity (mild, severe, serious) were recorded. A serious adverse event was defined as an event that resulted in death, was life-threatening, resulted in hospitalisation or prolonged hospitalisation, resulted in permanent or clinically significant disability or incapacity, resulted in a congenital anomaly or birth defect, or was considered serious for any other reason. A severe adverse event is defined as an event that causes an inability to work or perform usual activities and leads to discontinuation of the drug. Mild adverse events were defined as adverse events that did not require discontinuation of the drug or that could be resumed with symptomatic treatments, even if the drug was discontinued [11].

The NTD dose, hospitalisation during treatment and its cause, death and its cause, and whether malignancy developed were recorded.

On the other hand, we divided the patients into two groups, SSc-ILD and other CTDs-ILD, and compared the efficacy and safety of NTD in SSc-ILD and other CTDs-ILD.

### Statistical analysis

The conformity of the continuous variables to the normal distribution was analysed using Kolmogorov–Smirnov and Shapiro–Wilk tests. Continuous variables were expressed as mean ± standard deviation (SD) or median (Q1-Q3) and categorical variables as n (%). The Paired Samples t Test was used for the dependent continuous variables with normal distribution, while the Wilcoxon's test was used for the variables without normal distribution. When comparing two groups, the Independent Samples t-Test was used for continuous variables with normal distribution. The Mann–Whitney U test was used for variables that were not normally distributed. The chi-square test was used for the comparison of categorical variables. The SPSS programme (IBM SPSS for Windows, Ver.28) was used to calculate the statistical data. *p* < 0.05 was accepted as the statistical significance level.

## Results

Our multicenter study included 66 patients diagnosed with CTD-ILD in whom NTD was initiated due to the development of PPF during follow-up. The mean age of our patients was 61.7 years (± SD: 11.6) and the majority were female (66.7%). When patients were evaluated for CTD diagnoses, the most common diagnosis was SSc (*n* = 35, 53.0%). The most common comorbidity was hypertension (*n* = 30, 45.5%). The clinical and demographic characteristics of the patients are shown in Table 1.

**Table 1:** Clinical features, demographic characteristics and immunosuppressive medications of CTD-ILD patients

Parameters	*n* = 66 (100%)
Age (years)	61.7 (± SD: 11.6)
Sex	
Female Male Smoking (active or former) Pack-years (cigarette) median (Q1-Q3)	44 (66.7) 22 (33.3) 22 (33.3) 0 (0–18.25)
Connective Tissue Disease	
SSc	35 (53.0)
RA	14 (21.2)
SJS	12 (18.2)
MCTD	3 (4.5)
IIM	1 (1.5)
UCTD	1 (1.5)
Comorbidities	38 (57.6)
Hypertension	30 (45.5)
Coronary artery disease	18 (27.3)
Diabetes mellitus	12 (18.2)
Pulmonary arterial hypertension	10 (15.2)
Medical therapies before nintedanib	66 (100)
Glucocorticoids	59 (89.4)
Mycophenolate mofetil	48 (72.7)
Cyclophosphamide	35 (53.0)
Azathiopurine	22 (33.3)
Rituximab	22 (33.3)
Methotrexate	18 (27.3)
Tocilizumab	1 (1.5)
Medical therapies in combination	66 (100)
Mycophenolate mofetil	46 (69.7)
Glucocorticoids	39 (59.1)
Rituximab	18 (27.3)
Azathiopurine	5 (7.6)
Methotrexate	4 (6.1)
Tocilizumab	4 (6.1)
Cyclophosphamide	3 (4.5)
Abatacept	2 (3.0)

*CTD-ILD* Connective Tissue Disease-Interstitial Lung Disease, *IIM* Idiopathic inflammatory myositis, *MCTD* mixed connective tissue disease, *Q1-Q3* Percentiles 25–75, *RA* rheumatoid arthritis, *SD* standard deviation, *SJS* Sjögren's syndrome, *SSc* systemic sclerosis, *UCTD* undifferentiated connective tissue disease. median *(Q1,Q3)* mean (± SD)

Among the immunosuppressive therapies used before the onset of NTD, the use of GCs (*n *= 59, 89.4%) was the most common. MMF was used in 48 patients (72.7%) and was one of the other commonly used agents. RTX (*n* = 22, 33.3%) was the most commonly used biological disease-modifying antirheumatic drug (bDMARD) (*n* = 23, 34.8%). When the immunosuppressive therapies used by the patients were reassessed after the start of NTD treatment, all patients used at least one immunosuppressive drug in combination with NTD. MMF (n = 46, 69.7%) was the most commonly used agent. The use of GCs (*n* = 39, 59.1%) was less common in combination therapy. RTX was also the most commonly used bDMARD in combination (*n* = 18, 27.3%). The immunosuppressive therapies used by patients prior to NTD treatment and in combination with NTD are listed in Table 1.

HRCT and PFTs were available in all patients (*n* = 66) in the 6 months prior to NTD treatment. Disease duration prior to PPF was 81.6 (± SD: 80.7) months. When the criteria for the diagnosis of PPF were evaluated, 63 (95.5%) patients had clinical progression, 56 (84.8%) patients had physiologic progression, and 46 (69.7%) patients had radiologic progression. HRCT imaging showed a UIP pattern in 78.8% (*n* = 52) of patients, the most common pattern of involvement. A possible UIP pattern was detected in four (6.1%) patients and a fibrotic NSIP pattern was present in 10 (15.2%) patients.

In the control evaluation of our study after 6 months, 46 (69.7%) patients were evaluated, as 10 patients discontinued treatment due to side effects and 10 patients had a shorter follow-up period than 6 months or had no follow-up PFTs. There was a decrease in %pFVC and %pDLCO values between baseline and month six, but this was not statistically significant. The change in %pFVC at month six was −0.7 (± SD:9.7) and the change in %pDLCO was −0.9 (± SD:7.0).

At the 12-month follow-up, 20 (30.3%) patients had ongoing follow-up and PFTs, whereas at the 18-month follow-up, nine (13.6%) patients had follow-up parameters and PFTs. When %pFVC and %pDLCO values at both 12 and 18 months were compared to %pFVC and %pDLCO values at baseline, stabilisation was again seen. PFTs values at baseline, 6 months, 12 months and 18 months and changes at follow-up are shown in Table 2.

**Table 2:** PFTs of CTD-ILD patients at baseline and follow-up

		*n* = 46			*n* = 20			*n *= 9	
	Baseline	6 months	*p*	Baseline	12 months	*p*	Baseline	18 months	*p*
%pFVC	65.50 (58,81)	64 (55,83)	0.320	61 (55,70)	54.5 (45,67)	0.085	65 (61,73)	62 (56,72)	0.090
Change of %pFVC		−0.7 (9.7)			−3.0 (−11,1)			−4.8 (8.0)	
%pDLCO	44.8 (16.0)	43.8 (17.5)	0.330	43.7 (17.9)	44.5 (21.7)	0.752	50.1 (18.1)	49.8 (19.4)	0.957
Change of %pDLCO		−0.9 (7.0)			−1.0 (−4,1)			−0.2 (11.8)	

*CTD-ILD* Connective Tissue Disease-Interstitial Lung Disease, *PFTs* pulmonary function tests, *pDLCO* predicted diffusion capacity of the lungs for carbon monoxide, *pFVC* predicted forced vital capacity, *Q1-Q3* Percentiles 25–75, *SD* standard deviation,* p* < 0.05:statistical significance level. median (Q1,Q3) mean (± SD)

A subgroup analysis was performed for SSc patients (*n* = 35). There were 25 patients with 6-month follow-up and 11 patients with 12-month follow-up. Since there were only three SSc patients with a follow-up after 18 months, the 18-month evaluation was not performed. In the SSc patients, we observed stabilisation of %pFVC and %pDLCO values at follow-up. The change in %pFVC at six months was 0.1 (± SD:10.2) and the change in %pDLCO was −1.0 (Q1:−4, Q3:2). At the twelfth month, the change in %pFVC was −3.3 (± SD:11.6) and the change in %pDLCO was 0.0 (Q1:−2, Q3:6) (Table 3).

**Table 3:** PFTs of SSc-ILD patients at baseline and follow-up

		*n* = 25			*n *= 11	
	Baseline	6 months	*p*	Baseline	12 months	*p*
%pFVC	62.0 (57,80)	66.8 (24.8)	0.590	62 (48,72)	60.0 (39,81)	0.413
Change of %pFVC		0.1 (10.2)			−3.3 (11.6)	
%pDLCO	43.0 (39,59)	41.0 (36,55)	0.352	39.0 (39,64)	53.3 (23.9)	0.406
Change of %pDLCO		−1.0 (−4,2)			0.0 (−2,6)	

*PFTs* pulmonary function tests, *pDLCO* predicted diffusion capacity of the lungs for carbon monoxide, *pFVC* predicted forced vital capacity, *Q1-Q3* Percentiles 25–75, *SSc-ILD* Systemic Sclerosis-Interstitial Lung Disease, *SD* standard deviation, pFVC, pDLCO,* p* < 0.05:statistical significance level. median (Q1,Q3) mean (± SD)

Initially, all patients received an NTD treatment dose of 150 mg twice daily, but in one patient the dose was changed to 150 mg once daily due to side effects. The duration of NTD treatment was 12.8 (± SD:9.6) months. The most common adverse effect after treatment was diarrhoea (n = 20, 30.3%). Patients who developed diarrhoea were recommended loperamide, and NTD treatment could be continued in 15 patients who responded. No serious adverse events occurred in any patient. In 10 patients, NTD treatment could not be continued due to severe adverse events (diarrhoea (*n* = 5), elevated liver enzymes (*n* = 2) and nausea (*n* = 3)) despite dose reduction and/or symptomatic treatment. One patient was diagnosed with lung cancer and one patient with lymphoma during follow-up. Adverse events, hospitalizations and causes of death during NTD treatment are listed in Table 4.

**Table 4:** Nintedanib side effects and safety data

Parameters	*n* = 66 (100%)
Adverse events Nausea Diarrhoea Hepatic enzyme elevation Weight loss Loss of appetite Severity of side effects Mild Severe Interruption of nintedanib treatment Discontinuation of nintedanib treatment Hospitalisation during treatment Respiratory reasons Non-respiratory reasons Death during treatment Respiratory infection/pneumonia Unknown	30 (45.5) 17 (25.8) 20 (30.3) 5 (7.6) 8 (12.1) 18 (27.3) 30 (45.5) 20 (30.3) 10 (15.2) 14 (21.2) 10 (15.2) 22 (33.3) 15 (22.7) 7 (10.6) 6 (9.1) 3 (4.5) 3 (4.5)

We also divided the patients into two groups, SSc-ILD and other CTDs-ILDs, and compared them in terms of all the parameters we assessed (Table 5). RA (*n* = 14, 45.2%) and SJS (*n *= 12, 38.7%) patients constituted the majority of patients in the other CTDs-ILD group (*n* = 31, 100%). The mean age of patients in the other CTDs-ILD group was 64.8 years (± SD:10.8), which was higher than that of the SSc-ILD group (59.0 ± SD: 11.8) (*p* = 0.044). The proportion of smokers and patients with diabetes mellitus (DM) among comorbidities was higher in the other CTDs-ILD group (p-values = 0.015 and 0.031, respectively). Regarding HRCT patterns, 25 (71.4%) patients in the SSc-ILD group had a UIP pattern, two (5.7%) patients had a possible UIP pattern and eight (22.9%) patients had a NSIP pattern. In the other CTDs-ILD group, the UIP pattern was observed in 27 (87.1%) patients, probable UIP and NSIP patterns were found in two (6.5%) patients each, and there was no significant difference between the groups. When we compared patients in both groups with respect to PPF criteria, no statistically significant difference was found either. The change in %pFVC values after 6 months (SSc-ILD: −2.4 ± SD:8, other CTDs-ILD:−3.7 ± SD:5.9, *p* = 0.498), the change in %pFVC values after 12 months (SSc-ILD: −4.8 ± SD:10. 8, other CTDs-ILD:−7.5 ± SD:12.7, *p* = 0.595) and the change in %pFVC values after 18 months (SSc-ILD:−2.6 ± SD:7.9, other CTDs-ILD:−7.7 ± SD:8.3, *p* = 0.376), there was no significant difference between the groups. Also when comparing the change in %pDLCO values after 6 months (SSc-ILD: −1 (Q1:−4.5, Q3:9.0), other CTDs-ILD: 2.5 (Q1:−9.5, Q3:4), p = 0.817), 12 months (SSc-ILD: 9.2 ± SD:14.9, other CTDs-ILD: 0.2 ± SD:13.9, *p* = 0.185) and 18 months (SSc-ILD: 3 ± SD:9.9, other CTDs-ILD: −4.2 ± SD:14.3, *p* = 0.399) showed no significant difference between the groups.

**Table 5:** Comparison of clinical characteristics, demographic data, immunosuppressive therapies and nintedanib side effects and safety data of SSc and other CTD patients

Parameters	SSc *n*=35 (100%)	Other CTDs *n*=31 (100%)	*p*
Age (years)	59.0 (±SD: 11.8)	64.8 (±SD:10.8)	***0.044***
Sex			
Female	28 (80.0)	16 (51.6)	*** 0.015***
Male	7 (20.0)	15 (48.4)	
Smoking (active or former)	7 (20.0)	15 (48.4)	***0.015***
Pack-years (cigarette) median (Q1–Q3)	0.0 (0.0–6.0)	12.5(0.0–33.0)	***0.002***
Comorbidities	20 (57.1)	18 (58.1)	0.940
Coronary artery disease	7 (20.0)	11 (35.5)	0.159
Hypertension	16 (45.7)	14 (45.2)	0.964
Diabetes mellitus	3 (8.6)	9 (29.0)	***0.031***
Pulmonary arterial hypertension	9 (25.7)	1 (3.2)	***0.011***
Disease duration before PPF (months)	93.6 (±SD:75.6)	68.1 (±SD:85.4)	0.204
Nintedanib treatment duration(months)	14.2 (±SD:11.1)	11.1 (±SD:7.6)	0.189
Medical therapies in combination			
Mycophenolate mofetil	28 (80.0)	18 (58.1)	0.053
Glucocorticoids	18 (51.4)	21 (67.7)	0.179
bDMARDs	12 (34.3)	12 (38.7)	0.709
Azathiopurine	5 (14.3)	0 (0.0)	***0.029***
Cyclophosphamide	1 (2.9)	2 (6.5)	0.597
Methotrexate	0 (0.0)	4 (12.9)	***0.044***
Adverse events	19 (54.3)	11 (35.5)	0.126
Nausea	10 (28.6)	7 (22.6)	0.579
Diarrhoea	13 (37.1)	7 (22.6)	0.199
Hepatic enzyme elevation	4 (11.4)	1 (3.2)	0.209
Weight loss	6 (17.1)	2 (6.4)	0.184
Loss of appetite	12 (34.3)	6 (19.3)	0.174
Severity of side effects			
Mild	13 (37.1)	7 (22.6)	0.199
Severe	6 (17.1)	4 (12.9)	0.632
Interruption of nintedanib treatment	7 (20.0)	7 (22.6)	0.798
Discontinuation of nintedanib treatment	7 (20.0)	3 (9.7)	0.243
Hospitalisation during treatment	12 (34.3)	10 (32.3)	0.862
Respiratory reasons	7 (20.0)	8 (25.8)	0.574
Non-respiratory reasons	5 (14.3)	2 (6.5)	0.302
Death during treatment	2 (5.7)	4 (12.9)	0.311

*bDMARDs* biological disease-modifying antirheumatic drugs; *PPF* Progressive Pulmonary Fibrosis; Q1–Q3, Percentiles 25–75; SD, standard deviation; SSc, systemic sclerosis; *p* < 0.05:statistical significance level. median (Q1,Q3), mean (±SD)

There was no statistically significant difference between the two groups in terms of safety data such as side effects after NTD treatment, severity of side effects, discontinuation or interruption of the drug, hospitalisation during treatment and exitus.

## Discussion

In our multicenter study, we presented 18-month real-world data of patients who were followed up with CTD-ILD and started NTD treatment due to PPF during follow-up. There is limited real-world data on the use of NTDs in CTD-ILD patients [15–18]. We could find only two studies with real-life data on CTD-ILD that used progressive fibrosis as an inclusion criteria. Among these studies, the study by Ushio et al.[18] included 26 patients and the study by Boutel et al.[15] only 21 patients. Our study included the largest number of patients (n = 66) with CTDs-ILD in whom NTDs were used for PPF. It is also the first study in which patients were divided into two groups, SSc-ILD and other CTDs-ILD, and compared with each other. According to our results, there was stabilisation in PFTs in CTD-ILD patients who started NTD treatment. In a sub-analysis for SSc patients, we found similar results. Although all patients combined NTD with at least one immunosuppressant, the proportion of patients who discontinued NTD treatment due to side effects was not higher than in randomised controlled trials [8–10]. When we divided the patients into SSc-ILD and other CTDs-ILD, we also found that NTD treatment was effective and safe in both the SSc group and the other CTDs-ILD group.

ILD remains an important cause of morbidity and mortality in the course of CTDs despite advances in therapeutic options. It is the leading cause of mortality in CTDs, particularly in SSc [5].

Our study showed a general stabilisation of %pFVC and %pDLCO levels in patients who had started NTD treatment for PPF over a long period of 18 months during the course of CTD-ILD. In the double-blind, randomised, placebo-controlled INBUILD study of NTD use in ILD with progressive fibrosis, a smaller annual decline in %pFVC was observed in the NTD group compared to patients receiving placebo over a 52-week period [12]. The results of our study are consistent with those of the INBUILD study. A single-centre study of 21 patients treated with NTD for progressive fibrosing CTD-ILD also provided similar real-world data [15]. ​Ushio et al. also reported that NTD treatment was effective in slowing disease progression in patients with progressive fibrosis with CTD-ILD [18].

The majority of our patients were SSc patients. Therefore, we performed a separate analysis of changes in SSc patients after 6 and 12 months of follow-up. The result of the analysis was a stabilisation of %pFVC and %pDLCO values in SSc patients after 6 and 12 months. In the randomised, placebo-controlled SENSCIS trial of NTD use in SSc-related ILD, the rate of decline in %pFVC over a 52-week period was lower in those receiving NTD than in those receiving placebo. In the SENSCIS study, 48.4% of patients also took MMF in combination with NTD [13]. In a multicenter study conducted in Italy with real-world data on the use of NTDs in SSc patients, 90 patients were examined. The study, which presented the results of 49 patients with six-month follow-up data and 40 patients with 12-month follow-up data, reported that lung function stabilised after NTD treatment. In this study, the rate of patients receiving combined immunosuppressive therapy with NTD was 86%, while the rate of patients receiving MMF was 81% [16]. The results of our study are consistent with these real-world data and the results of the SENSCIS study. On the other hand, in contrast to these studies, all our patients received at least one immunosuppressive treatment in combination with NTD. The rates of GCs, methotrexate (MTX), AZT and CYC in combination with NTD treatment were decreasing. One of the main factors for these results is that MTX treatment, although rarely associated with idiosyncratic pneumonia, has been modified by clinicians due to insufficient evidence of its benefit in the treatment of ILD. AZT treatment can also be used as first-line therapy, but is usually considered an additional option due to limited evidence of its efficacy in SSc-ILD [9]. As the majority of our patients were SSc-ILD patients and progression was observed, AZT treatment was reduced. The most important factor for the decrease in the use of CYC is the thought that the adverse events might be higher compared to other agents [9]. Therefore, drug therapies such as MMF or RTX are preferred, especially for long-term treatment. On the other hand, we often use GCs at lower doses in this group of patients, particularly because of the risk of renal crisis that can develop during the course of SSc. However, the decrease in the use of GCs was remarkable, especially after treatment with NTD.

The incidence of adverse events in our patients (45.5%) is lower than in randomised controlled trials [12, 13]. The most common side effect was gastrointestinal side effects, among which diarrhoea was the most common. Our findings on gastrointestinal side effects were consistent with the results of randomised controlled trials and studies with real-world data [11–13, 16, 19]. The proportion of patients who were unable to continue treatment despite dose reduction (15.2%) was generally not higher than in randomised controlled trials [12, 13]. Both of our patients who were diagnosed with lung cancer and lymphoma during follow-up were SSc patients. We know that the risk of malignant disease, especially lung cancer, increases during the course of SSc [20]. No NTD-related side effects were observed in our three patients with respiratory tract infection/pneumonia as the cause of exitus. Immunosuppressive therapies are frequently used in both ILD and non-ILD involvement in CTD [21]. In the INBUILD study, patients receiving combination therapy were excluded, but after 6 months, additional immunosuppressive therapy was allowed in patients who needed it [12]. In the SENSCIS study, patients who received MMF were also included in the study [13]. In contrast to randomised controlled trials, the treatment of NTD is often combined with immunosuppressants in studies with real-world data [15, 16]. Although all our patients took the NTD in combination with at least one immunosuppressive therapy, there was no significant increase in the discontinuation rate due to an adverse effect. At the same time, gastrointestinal side effects were similar to those reported in the literature despite the use of MMF. On the other hand, the proportion of patients who used a combination with RTX (27.3%) was significantly higher. The evaluation of all these results leads to the conclusion that the combined use of NTD treatment with immunosuppressants does not raise additional safety concerns.

Studies on the use of NTD in CTD-ILD are limited. In SSc-ILD patients, both the randomised, placebo-controlled SENSCIS trial and the multicenter trial with real-world data concluded that NTD use is effective and safe [13, 16]. In a study with real-world data from RA-ILD patients, both NTD and pirfenidone use were evaluated. The results of this study show that both antifibrotics lead to a decrease in %pFVC and the most common side effects are gastrointestinal side effects [17]. As far as we know, there are only two studies with real-world data on the use of NTDs in CTD-ILD patients [15, 18]. In one of these studies, 11 out of a total of 26 patients were SSc patients and in the other study, 9 out of a total of 21 patients were SSc patients. We divided the patients in our study into two groups: other CTDs-ILD (*n* = 31) and SSc-ILD (*n* = 35) and compared them with each other. Although the patients in the other CTDs-ILD group were older and had higher rates of DM, smoking and comorbidities, we found similar safety data to the SSc-ILD group. On the other hand, there was no significant difference between the two groups in the change in %pFVC and the change in %pDLCO values. Regarding HRCT patterns, although the NSIP pattern is expected to be more common in SSc-ILD patients [4], the main reason for the higher frequency of the UIP pattern in our patients is that we performed NTD treatment in patients with fibrosis. Among our patients in the other CTDs group, there were a considerable number of SJS patients in addition to RA.

When we evaluate the strengths of our study, in contrast to the randomised controlled INBUILD and SENSCIS studies [12, 13] and the few SSc-ILD and RA-ILD real-life studies [16, 17], all our patients receive immunosuppressive treatment in addition to NTD treatment. Despite this situation, NTD treatment was safe. On the other hand, the use of NTD was also effective and safe in patients with other CTD-ILD, such as SSc-ILD.

The main limitation of our study is the lack of a control group, as it is a restrospective study. Another limitation is the small number of patients, especially in the group of other CTDs. The most important factor leading to this situation is that these patients were not included in the study because the PFT results required for follow-up at 6, 12 and 18 months were missing.

## Conclusion

In summary, the use of NTD in combination with immunosuppressive therapies was effective and safe in patients with SSc-ILD as well as in patients with other CTDs-ILD. More real-world data and randomised controlled trials on the efficacy and safety of NTD need to be planned, especially in patients with CTDs-ILD other than SSC-ILD and RA-ILD. On the other hand, the efficacy and safety of NTD in combination with immunosuppressive therapy are among the unmet needs.

## Data Availability

The data will be provided by the authors without hesitation if requested.
